# Recent Progress toward Engineering HIV-1-Specific Neutralizing Monoclonal Antibodies

**DOI:** 10.3389/fimmu.2016.00391

**Published:** 2016-09-30

**Authors:** Ming Sun, Yue Li, Huiwen Zheng, Yiming Shao

**Affiliations:** ^1^Institute of Medical Biology, Peking Union Medical College, Chinese Academy of Medical Sciences, Kunming, China; ^2^State Key Laboratory of Medicinal Chemical Biology, College of Life Sciences, Nankai University, Tianjin, China; ^3^State Key Laboratory for Infectious Disease Prevention and Control, National Center for AIDS/STD Control and Prevention, Chinese Center for Disease Control and Prevention, Beijing, China; ^4^Collaborative Innovation Center for Diagnosis and Treatment of Infectious Diseases, Zhejiang University, China; ^5^School of Medicine, Nankai University, Tianjin, China

**Keywords:** HIV-1 neutralizing antibodies, antibody engineering, bispecific antibody, variable region, antigen binding, gene delivery

## Abstract

The recent discoveries of broadly potent neutralizing human monoclonal antibodies represent a new generation of antiretrovirals for the treatment and prophylaxis. Antibodies are generally considered more effective and safer and have been proved to provide passive protection against mucosal challenge in humanized mice and macaques. Several neutralizing Abs could protect animals against HIV-1 but are not effective when used in an established infected model for therapy. In order to overcome the limitation of antiviral activities, multiple antibody-engineering technologies have been explored to generate “the better” neutralizing antibodies against HIV-1 since bNAbs attack viral entry by various mechanisms. Thus, a promising direction of research is to discover and exploit rational antibody combination or engineered antibodies (eAbs) as potential candidate therapeutics against HIV-1. It has been reported that inclusion of fusion-neutralizing antibodies in a set of bNAbs could improve their overall activities and neutralizing spectrum. Here, we review several routes for engineering bNAbs, such as design and generation of bispecific antibodies, specific glycosylation of antibodies to enhance antiviral activity, and variable region-specific modification guided by structure and computer, as well as reviewing antibody-delivery technologies by non-viral vector, viral vector, and human hematopoietic stem/progenitor cells transduced with a lentiviral construct. We also discuss the optimized antiviral activities and benefits of these strategy and potential mechanisms.

## Introduction

Currently, through single memory B-cell sorting from HIV-1-infected patients and micro-neutralization screening of B-cell cultures, many new monoclonal antibodies (mAbs) have been isolated and characterized successfully, such as VRC01, PG9, 3BCN117, NIH45–46, PGT Abs, and 10E8 (1–5). Previously, it was a concern that an increase in the antiviral breadth may be accompanied by a loss in neutralizing activity, but new broadly neutralizing antibody (bNAbs) can offer potent and broad neutralization of diverse HIV-1 isolates (6). These discoveries have led to further optimism that effective vaccines against HIV-1 will be discovered, especially those that induce cross-clade neutralizing antibodies (7). However, how to translate the new knowledge about neutralizing epitopes into immunogens that elicit potent and persistent immunity is still a challenge (8).

Since the new-generation antibodies exhibit a sufficient level of breadth in their ability to neutralize genetically diverse HIV-1 strains, as an alternative approach, passive immunization using broadly neutralizing antibodies can be applied for immunization and therapy for HIV-1. Several broadly neutralizing mAbs have been shown to protect against HIV-1 infection in animal models when administered as monotherapy (9–12). However, as a threat, viral resistance is still present in HIV-1 clinical therapeutics, and HIV-1 has been shown to develop resistance to neutralizing antibodies as well. By now, it has been reported that antiviral activity of new-generation neutralizing antibodies in treating an established HIV-1 infection is limited (10, 11, 13–15). Particularly, viral load rebounds quickly in all infected patients treating either high or low doses of neutralizing therapeutic antibody due to the outgrowth of pre-existing or *de novo* viral escape variants. Thus, it is very difficult for antibodies to eliminate the virus and virus-infected cells *in vivo* once the resistant mutants rapidly emerge.

On the other hand, there is no cross-resistance with the currently feasible antiretroviral to give their distinct mechanisms of antiviral action. It has been reported that combining several different antiviral mechanism’s neutralizing antibodies would increase the barrier against resistance *in vivo* than any one antibody alone (10, 11, 13, 16–18). Thus, novel broadly potent neutralizing antibodies are needed to deter the development of HIV-1 resistance.

Parent neutralizing antibodies can be engineered to improve their antigen-binding affinity, effect functions, improve their half life, and apply new delivery methods *in vivo*. Thanks to advances in antibody-engineering technology (19, 20), such as variable-region modification, modification of the glycosylation pattern, and design of bispecific antibody (bsAb) formats, it has been possible to create novel optimized mAbs that may improve the antiviral activity of bNAbs or target multiple viral epitopes to overcome neutralization escape by potency and breadth improvement. In addition, using viral vectors or non-viral vector-mediated technology to deliver the antibodies into circulation, or even implantation of an HIV-specific bNAb gene into hematopoietic stem/progenitor cells (HSPCs), can provide anti-HIV mucosal immunity by effectively reprogramming the immune system. These new platforms for engineering antibodies make HIV-1-neutralizing antibodies more available as candidate therapeutics against this virus.

## Broadly Neutralizing Antibodies Against HIV-1

**Figure 1:**
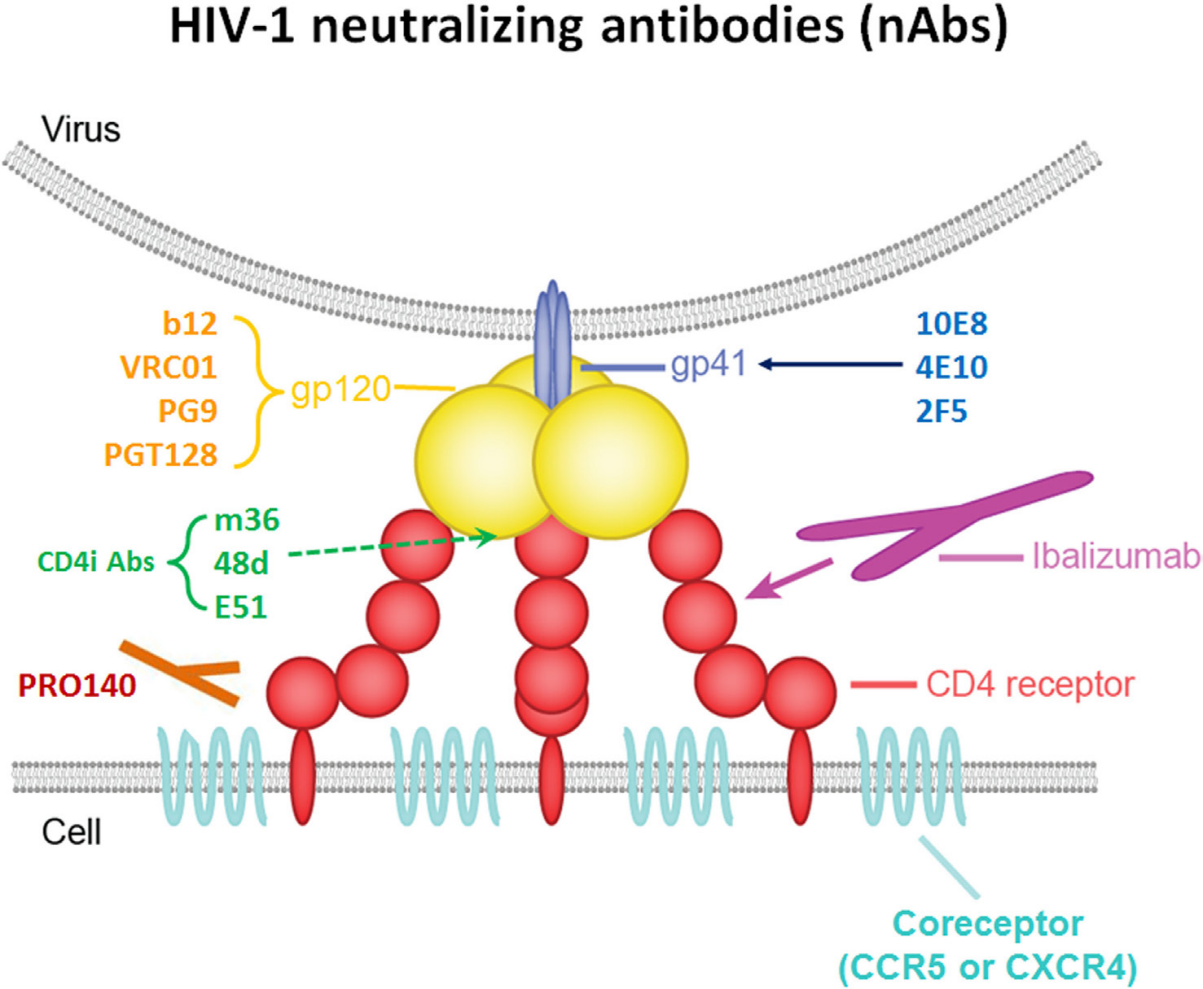
**Schematic interactions between HIV-1 and a T cell in the presence of broadly neutralizing antibodies (bNAbs)**.

HIV-1 entry into cell is triggered by interaction of the viral trimeric envelope (Env) glycoprotein gp120 with CD4 in T cell surface (21, 22). The key steps include (A) binding of gp120 to its receptor CD4 on T helper cells, with the formation and exposure of the coreceptor (CoR)-binding site in gp120; (B) binding of the HIV-1’s gp120 to CoRs (CCR5 or CXCR4); (C) fusion of the viral and host cellular membranes (21, 23). Therefore, HIV-1 infection elicits specific antibody against both gp120, gp41 of the Env and conformational epitopes induced by trimmer. Meanwhile, viral entry makes targets available for new-generation neutralizing antibodies to inhibit HIV-1 infection (Figure 1).

Broad and potent anti-HIV-1 neutralizing mAbs can inhibit entry of the virus into the host cell by various mechanisms. One mechanism is interfering with the attachment of the virus to host cell surface receptors. This can be achieved when the antibody binds to gp120 on HIV-1 spikes, thereby interfering with the binding of the virus’s gp120 to the host cell’s CD4 (24). The same inhibitory effect is achieved by antibodies targeting T cell receptor CD4 or CoRs, such as CCR5 and CXCR4, thereby interfering with virus interaction with cell membrane or blocking the receptor binding sites for the virus unavailable, resulted in against HIV-1 entry (25). Another neutralizing mechanism is post-attachment/pre-fusion neutralization and interference with gp120-required conformational changes and fusion processes at the target cell membrane.

Several specific sites on gp120 have been shown to be targeted by bNAbs. (A) The CD4-binding site (CD4bs) on gp120 is recognized by a number of broadly neutralizing mAbs. These Abs include VRC01, NIH45–46, 12A12, and 3BNC117 (1, 4, 8, 26–28), which neutralize 80–90% of circulating HIV-1 isolates with high potency *in vitro*, as well as CH103 and 8ANC131 with relatively less potency; (B) the V1V2 site has been proven to be another site of the spike that is vulnerable to neutralizing antibodies. PG9, PG16, and PGDM1004 are representatives of V1V2-directed neutralizing mAbs that generally compete with one another for antigen (29–31); (C) N-linked glycan-containing epitopes form a site of spike vulnerability, including the gp120 V3 loop, and several nearby glycans, 2G12, PGT128, and PGT121/10-1074 represent the bNAbs that recognize this region (2, 32, 33); (D) there are also bNAbs that target the coreceptor-binding site (CoRbs) on gp120. The exposure of this site, also known as the CD4-induced (CD4i) site, is (as its name suggests) induced by conformational changes that occur as a result of the binding of CD4 to gp120. Antibodies targeting this region are called CD4-induced antibodies, and include 17b, X5, m36, 48d, and E51 (34–36). Based on the length of the IgG heavy chain CDR, most full-size CD4i Abs do not have high antiviral potency because full-size Abs targeting CD4i-specific epitope is sterically restricted during viral entry (37).

The membrane-proximal external region (MPER) of gp41 contains highly conserved residues that also play a critical role in the virus fusion process. Only 671–683 AA as a linear epitopes of this MPER are recognized by potent neutralizing mAbs (38, 39). These bNAbs include 4E10, 2F5, Z13, and 10E8 (3, 40, 41). Although previous studies show that combination of 2F5 and 10E8 protects rhesus against SIV infection, 10E8 still exhibits most potent and broad neutralizing activity compare with other MERP active antibody targeting gp41 of HIV-1. It can neutralize about 92% of circulating HIV-1 isolates *in vitro*.

Ibalizumab (iMab) is a mAb that has broad and potent activity against HIV-1; it inhibits HIV-1 by binding mainly to domain 2 (D2) of CD4 on host target cells, inhibiting post-CD4 binding events required to infect cells (42). PRO-140 is a monoclonal antibody directed against the CCR5 CoR (CCR5 antagonists), and it potently inhibits CCR5-mediated HIV-1 entry without blocking the natural activity of CCR5 *in vitro* (43).

Almost all of the potent neutralizing antibodies have some unusual features. The CD4-specific antibodies exhibit unusual maturation with somatic maturation frequencies of 20–32%. The V1V2-specific antibodies display unusually long CDRH3 of 24–32 amino acid. The V3 glycan-specific antibodies show a relatively high somatic mutation CDR H3s with deletions or insertions. The gp41’s MPER-specific antibodies display relatively high somatic mutation. Understanding the targeting sites, inhibition activities, and unusual feature of above antibodies will contribute to generation of more potent neutralizers by antibody engineering.

## HIV-1 Neutralizing Antibody Engineering

As we know, the Env-targeted antibodies comprise mainly of binding antibodies without neutralizing activity or strain-specific antibodies after HIV-1 infection or HIV-1 vaccine immunization. The hurdles of neutralizing antibody generation against HIV-1 are mainly caused by conformational mask and diversity of HIV-1 strains. The new-generation bNAbs, being applied for therapy, also faced the limitation of antiviral breadth and potency. Thus, antibody engineering might provide a potential approach to overcome the limitation. The primary role of the antibody’s variable region is to bind to the specific antigen or epitope, resulting in neutralization against HIV-1. The affinity and the specificity of the mAb are two important antigen-binding properties, and engineering these properties has been extensively studied recently to improve the effectiveness of HIV-1 neutralizing antibodies. Meanwhile, new strategies are currently focused on adding more functionality to existing mAbs. That is, there are a variety of strategies being undertaken to enhance the antiviral properties of mAbs.

### Bispecific Antibody Design and Mechanism

At present, although using monotherapy by neutralizing antibodies can protect against HIV-1 infection in animal models, the efficacy in administrating an established infection is not satisfied. One of the reasons is that HIV-1 strains’ RNA polymerases are devoid of proofreading and repair capabilities, resulting in the emergence of resistant mutants under neutralizing antibody selective pressure. Furthermore, several viruses treated with a non-specific antibody have been shown to generate the mutated residues associated with viral escape, suggesting minor pre-existing virus was amplified (44). Recent studies showed that although such mutations may produce an escape resistance to an HIV-specific bNAb, treatment of HIV-1 infected humans with a monoclonal antibody transiently reduced viral loads (14, 15). In a follow on study, it was shown that after treatment with a MAb, patient serum displayed enhanced neutralizing breadth, but the virus rebounded and certainly was not cleared (14). As we know, “people don’t make monoclonal antibodies” when they are infected with a pathogen. In other words, using polyclonal antibodies, several mAbs in combination or a bsAb can imitate the methods by which immunoglobulins *in vivo* clear or block pathogens. Thus, strategies of using several bNAbs in combination, and especially of using the synergistic antiviral activity of bispecific antibodies against HIV-1 have been explored in order to confront the emergence of resistant mutants.

**Figure 2:**
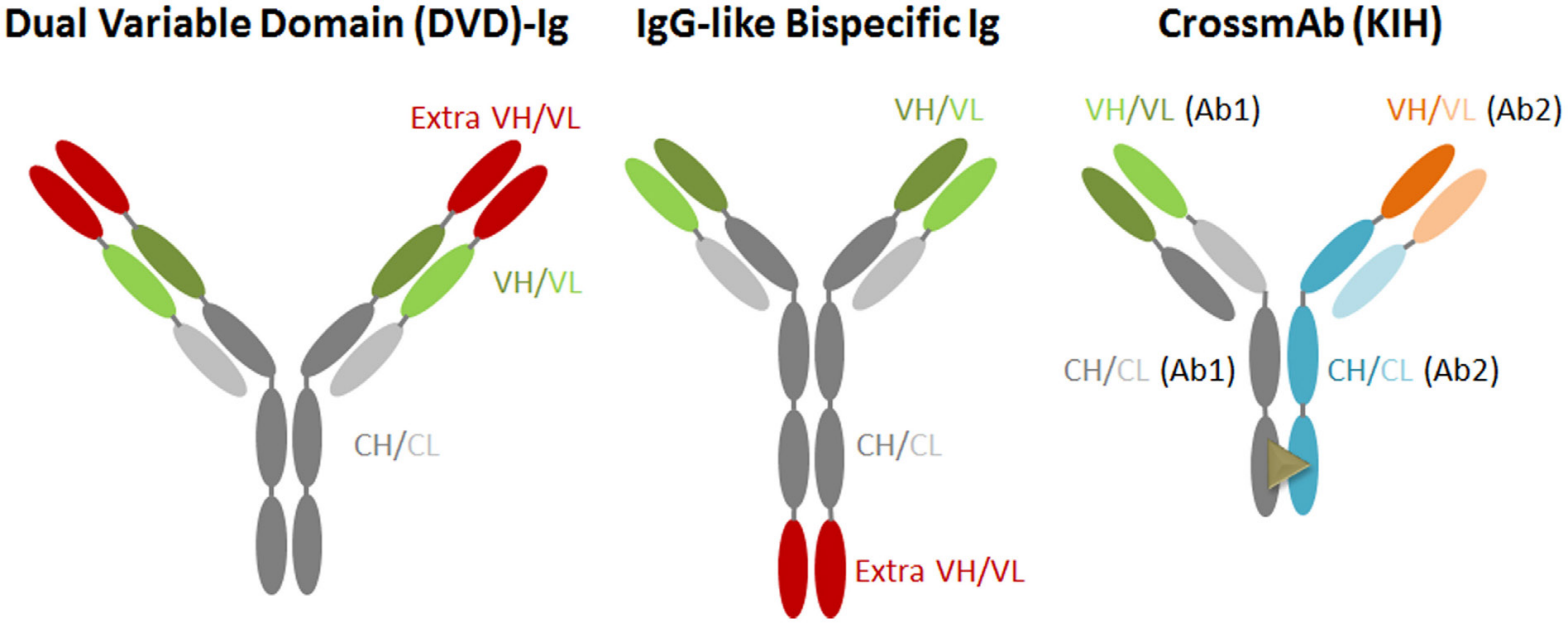
**Schematics of engineered antibodies for anti-HIV-1 investigations**.

Novel antibody-engineering technologies have led to the generation of antibodies with amounts of different shapes and sizes, including bispecific and multi-antibodies. The bsAb against two targets represents one of the promising new-generation antiviral therapeutic antibodies. This type of antibody targets two different antigens or epitopes on the pathogen with different inhibitory actions, or could link cells to create an immune response (Figure 2). Generally, it is designed in accordance with several main criteria: (A) the candidate mAbs should process an antiviral breadth attacking distinct epitopes and non-overlapping; (B) the candidate mAbs should not compete with each other for antigen binding; (C) if possible, resistant HIV-1 mutants picked from one mAb would be neutralized by another non-selecting mAb; (D) selected antibody should exhibit bivalent binding against the virion, and with a special geometry that overcomes the low concentration of spikes on the HIV-1 surface; (E) the fused antibody should be of low immunogenicity; and (F) the antibody should be able to be purified simply by affinity for a protein A/G or by other chromatographic methods.

Currently, various bsAb versions, such as IgG-like bispecific antibody (IgG-scFv) and dual variable domain immunoglobulin (DVD), have been designed and generated (45, 46). The IgG-scFv is usually designed and expressed by fusing another antibody’s scFv to the heavy chain of IgG’s C or N-terminus *via* a flexible GS linker peptide; this bsAb keeps the original IgG constant domains intact, and, thus, exhibits crystallizable fragment (Fc)-mediated activities. CrossMab is also an IgG-scFv generated by the association of the heavy chains using the “knobs into holes” (KIH) approach; this approach is based on single amino acid residue substitutions in the opposite CH3 domains, and thereby promotes heavy chain heterodimerization (47, 48). In one of the heavy chains referred to as a “knob” variant, a small amino acid residue is replaced with a larger one (T366Y), and, in the other CH3 domain, a large amino acid residue is replaced with a smaller one (Y407T). It provides different heavy chain interaction with the “knobs” variant mimicking a key-lock concept. As a bsAb, CrossMab is a monovalent antibody format but is less immunogenic compared with IgG fusion bsAb.

In 2013, Pace et al. created IgG-like bispecific Abs that combined inhibition of the attachment of HIV-1 virus to the host cell surface by a mAb directed against CD4, with that of anti-gp120 with two copies of pG9 scFV; these antibodies exhibited potency and breadth, neutralizing most of the 118 HIV-1 strains tested at very low antibody concentrations (49). Another type of bsAb combines CD4 and CD4i antibodies, and also functioned against HIV-1 in several studies. It is a fusion protein of domains 1 and 2 (D1D2) of soluble CD4 (sCD4) with 17b, and it exhibited broader and more potent activity than did b12, 4E10, and 2G12 in many neutralizing HIV-1 strains (50).

CrossMab of PRO140/10E8 and iMab/10E8 are designed based on different neutralization mechanisms, one of which involves inhibition of virus attachment to CD4/CCR5 of the host cell surface; the other in a pre-fusion neutralization manner. It also improves the antiviral activity significantly at a potency about 20-fold higher than that of the parent antibody by anchoring 10E8 at the site of viral entry into host cell, enhancing both the local antibodies’ concentration and accessibility to their cognate epitope (51). Notably, the activities of the PRO140/PGT128 and PRO140/117 crossMabs do not compare with that of the parent antibody due to monovalent binding. The Bill and Melinda Gates Foundation Collaboration for AIDS Vaccine Discovery (BMGF CAVD) has now selected this crossMab formatted bispecific IgG for the new generation of preventive and therapeutic candidates for HIV/AIDS.

### Variable Region Modification

Although the variable region is responsible for the antigen-binding properties of neutralizing antibodies, it can also affect their immunogenicity. It has been noted that antiviral activity of one of the broadly neutralizing HIV-1 antibodies, NIH45–46, was improved significantly by introducing a G54W mutation into the CDR2 in the heavy chain variable region using structure-based design (52). Since the CD4-binding site of gp120 is a vital target and recognized by NIH45–46, random modification of the CDRH2 of antibody might, even though slightly, increase the binding energy between the bNAb and gp120, and subsequently destroy the interaction between antibody and antigen, leading to loss of bNAb antiviral activity. However, in this case, structure-based design approaches to rationally modify the residues of the NIH45–46 heavy chain variable region that contact gp120 partly mimic approaches to mAb affinity maturation and simplify the procedure of maturation without a relative change in immunogenicity.

Recently, Song et al. improved one neutralizing mAb’s inhibition of HIV-1 viral activity by carrying out a strategic and specific glycosylation of the antibody (53). In this study, based on activity of ibalizumab against 118 HIV-1 strains and structural modeling, a 9mer glycan on the N-terminus of gp120 fills a vacant space between the L chain’s variable region of ibalizumab and gp120 V5. Therefore, potential N-linked glycosylation sites at various positions in the L chain were created in this mAb. One particular mutant (LM52) neutralized almost 100% of the tested 118 HIV-1strains at potency more than 10-fold higher than that of the parental antibody. Indeed, strategically adding a glycan into the antibody is more reasonable than introducing an artificial loop with a different length because glycan is typically non-immunogenic compared with protein. Besides, all modifications focused on a position of the light chain close to gp120 V5, and engineered glycosylation of the antibody minimizes the influence of the interaction between light chain and gp120.

Engineering efforts are also currently aimed at modifying antibody constant regions since neutralizing antibodies can also exert anti-HIV-1 effects, such as antibody-dependent cellular cytotoxicity (ADCC) and antibody-dependent cell-mediated virus inhibition (ADCVI), using the Fc portion of the full-length antibody (54–56).

### Broadly Neutralizing Antibody Delivery *In Vivo*

Although several broad and potent neutralizing antibodies have been isolated recently, an unresolved key issue is why only certain infected patients produce bNAbs against HIV-1. One of the explanations is that the ability to generate bNAbs is determined by host genetic factors, but there are not enough studies to support this hypothesis. Since it is still a challenge to elicit immune response *in vivo* against HIV isolates, passive infusion or antibody gene delivery might be of use. However, passive infusion also has several shortcomings, particularly in a prophylactic setting. These mAbs have a limited life span, and they need to be injected repeatedly over a long period of time, and repeated dosing carries a risk of injection-associated infection (57, 58). Thus, passive infusion as a prevention regimen on a large scale is impractical.

Gene transfer, one potential approach to overcome such limitations, requires the development of efficient and non-toxic polynucleotide delivery mechanisms (59–61). Muscle cells are capable of synthesizing, secreting, and generating a properly folded antibody; hence, antibody gene transfer *via* electroporation (EP) into muscle cells has great potential in terms of therapeutic applications (62, 63). Remarkably, the antibody concentration obtained *in vivo* in our studies is at least 10-fold higher than what is required to neutralize a large panel of viruses up to 100% efficiency by novel engineered Abs (64).

Vector-mediated gene transfer could be used to engineer secretion of broadly neutralizing antibodies *in vivo*. Johnson et al. have successfully used a gene transfer approach to produce *in vivo* an SIV-neutralizing monoclonal antibody by intramuscular (IM) injection of a recombinant adeno-associated virus (rAAV) vector carrying the bNAb gene (65). Notably, the antibody concentrations measured in the serum were well above the IC_50_ required for SIV neutralization for many months after inoculation. Most importantly, these monkeys were also protected from SIV infection (66). Meanwhile, the eCD4–IgG studies also display remarkable protection against SHIV challenge in macaques by AAV-expressed eCD4–Ig (67).

The use of AAV-based vectors has been reported to result in long-lived expression of full-length human antibodies in muscle after a single intramuscular injection that could fully protect humanized mice from HIV infection (13). In this study, b12–IgG serum concentrations of 100 mg/mL in humanized mouse after rAAV injection could protect from HIV-1 infection at challenge doses 100-fold higher compared with necessary doses to infect the non-human primate. In 2012, the same lab provided human HSPCs transduced with a lentiviral construct encoding b12–IgA2 into the humanized bone marrow-liver-thymus (BLT) mouse (68, 69). The humanized mice with a very low b12–IgA concentration in plasma and mucosal sites were fully resistant to mucosal challenge with HIV-1.

Advances of engineered humoral immunity with potent neutralizing anti-HIV antibodies using delivery of different antibody genes can effectively reduce disease progression. And these novel antibody delivery approaches would be even more beneficial if they are applied combining with those recently discovered new generation potent broadly neutralizing antibodies.

## Understanding Engineered HIV-1-Neutralizing Antibodies

For HIV-1 neutralization, there are several reasons why it is difficult to find targets for bNAbs (8, 70, 71), including (A) complex genetic diversity of HIV-1 clades, circulating recombinants and mutants; (B) that glycans of gp120 cover the neutralizing epitopes, which makes them inaccessible to antibodies; and (C) that HIV-1 all strains bind to the CD4 molecule, but the CD4-binding site of gp120 is located in a pocket that is difficult for an antibody to access. Thus, optimized activities by HIV-1 neutralizing antibody engineering display one of the potential approaches to solve the above difficulties.

In general, virus neutralization is considered to happen when a neutralizing antibody occupies the adequate epitopes on the viral surface. This “occupancy” model, also known as the “multi-hit model,” indicates that, when neutralizing antibodies achieve enough binding density on a virion, it will result in inhibition of attachment to cellular receptors or fusion processes (72). Here, engineering the antigen-binding properties of neutralizing antibodies provide better inhibitory activity with the “critical binding site” model compare with “multi-hit” model. High affinity/specificity modification, the potent neutralization is determined by its targeting of vital binding sites and be less dependent on achieving high antibody densities against HIV-1. Moreover, engineering antibodies may enable these optimized potent antibodies to be delivered at a lower dose with less frequency *in vivo*.

In terms of synergistic antiviral activity of bispecific antibodies, binding with higher affinity usually contributes to neutralizing activity of viruses expressing high densities of spikes (73). However, HIV-1 has a limited number of spikes and low density of viral trimers will cause neutralizing antibody to be less efficient for inhibition through interfering with bivalent binding to the HIV-1. Mature HIV-1 particles express only 10–15 randomly distributed viral spikes, and the spikes are, thus, spaced too far apart to be bridged by a bivalent antibody (73, 74), suggesting that only the special geometry of a bsAb can overcome the low density of spikes on HIV-1. Thus, bivalent binding needs rational design to generate heterotypic bivalent (bispecific) binding for HIV-1, such as pro140/10E8 CrossMab. In a word, dual conserved targets of bispecific antibodies diminish the importance of steric hindrance to access the tight crypt during viral entry into cells. The bispecific antibodies also mediate the cross-linking of virions that, when combined with greater activity, results in inhibiting HIV-1 entry into host cells.

For variable region modification, the targeted mutagenesis approach utilizes site-directed mutagenesis against the functional contact region to generate limited collections of the specific variants of the parent antibody. Screening based on divergent HIV-1 Envs could reduce the cross-reactivity to other antigens, broaden the specificity to HIV-1 cognate antigens, overcome genetic diversity of HIV-1, and substantially enhance its inhibitory activity. Meanwhile, the effect of glycan has been studied, and it was found that Fc bearing fucosylated and sialylated glycans have reduced affinities for FcRs (75). It has been reported that an N-linked carbohydrate specific introduction into IgG’s variable region caused optimized solubility (76, 77). Optimized placement of glycan in the antibody variable region, using the steric hindrance against viral entry strategy, markedly improves the antibody’s activity. Indeed, the precise and proper glycan-addition approach may be applied to enhance other bNAbs’ functional activity against HIV-1. As a potential alternative approach, HIV-1 antibody gene delivery technology provides the possibility of high density that antibodies bind to virion through relative stable and persistent expression, resulting in protection against HIV-1 mucosal infection.

HIV-1 presents special hurdles to producing neutralizing antibodies with high potency and breadth. In order to successfully defend against HIV-1, extensive strategies may be required to generate “the better” antibody against virus by applying multiple novel antibody engineering technologies. Variable region technologies, bsAb generation, and antibody delivery would appear to be essential technologies for developing next-generation antibody therapeutics to enhance the magic bullet with radiation against HIV-1.

## Author Contributions

MS and YL were involved in the review design and writing of the manuscript. HZ performed figure drawing of the manuscript. YS was involved in the manuscript drafting and review for contribution of knowledge and correction, as well as the final draft approval.

## Conflict of Interest Statement

The authors declare that the research was conducted in the absence of any commercial or financial relationships that could be construed as a potential conflict of interest.
